# Supplementary material for: Parallel visual circuitry in a basal chordate
Source: eLife. 2019 Apr 18;8:e44753. doi: 10.7554/eLife.44753 (PMC6499539; doi:10.7554/eLife.44753)
Supplement: Supplementary file 1. [file elife-44753-supp1.docx]

>CiVGLUT

atgtcaaacaataataaggagggaggttttgcgacggcatcacatgaccttctcagaacaattcaaaacgggtttcgaagtgttttatataggttcaccggaatcggcaaccctccataccaggaacaaactttggacagaaactcaagtttgaatgagatttcacgcaattcccaagagcatggggaagacacctccgaagaggacgtcccacaaacgaacaacaagtggatgtggtatgcaatgtgtctctggttacccaaacgttacgtgatggctttcttgagtgggtttggtttttgcatcacttttggcatgcgatgtaacctgggcgtggcaatggtcgaaatggcaaacaactacaccgaaacgttggaaaatggaacgaaagttattatgccacccgacttgtcatggtcctcagaaaaacaaggtttcattcacgggtcgttcttctggggttatataataactcaagtcccaggcggttacttggcttcccgcctcagagcgaacagggtgtttggcgtggcgatcctctgtacctgtttactcaacatgtttctgcccgctgctgccaaagcacattgggttgtcttcgtcatcgtacgtgttatgcaaggactagctgagggtgttttgtacccatcatgtcacgggatttggagcaaatgggcgccgcctttagaaaggagcagactagctaccatctcgttttcgggtagctacgctggtgctgtgatcggaatgccaatcgggggaatgttggtcgaatacgcaggatggccttctgtattttatgttttcggcagttgtggtattgcctggttcttcttatggacattcacctcatacgactcccccgcatctcacccacacataaggagaagtgaacgcatttacattgaagaaagtatcggcaagtcggactgtgcaactataccaatggtgagaacattatttaacaaattaggaacaccttggaagaaattcttgacctccctcccagtatgggcgattatcgttgctaacttctgcagaagctggaccttctatcttctaattatcagccaacccgcttattttgagcaagtcctaaaatatgacatttcacagcagcttggattcctcgcagccgttcctcacttggtgatgacgattatagttccgtttggtggcgtgcttgctgactttctgcgaaaaaaagaaatcttatcaaccaccaatgttagaaaagtaatgaactgtgggggtttcgggatggaggcgttgtttcttcttatccttgcttgcagtcatggacataccgctgctgtggtgtgcctggtgttcgccgtcggtttcagtggattcgccatctcaggtttcaacgtgaaccacctagatattgctccgagatacgcgagtatattgatgggcctttccaatggcgcagggacactgtcagggatgatatgtcctctcctggtcagctacattacacgtacaaagacggaggaagactggaaagtggtgttcgtgattgcatcatgcattcacttcagtggagtcatattttacgctttctttgcatctggtgagaggcaaccatgggccgacccaccccaggaagaagttggtattttagacggggaaaacttcgccgcttccccgtcaatgacctcgtttcgtcgtcgatccggtatgtcgcgcggatcttcctcctcggattcgatgttcggcgagggtccggatgacctgtatgtaaagaaacttacgaacggtgtctacaatccgaatttcgagggaggacctcggagtaaaagattcagcaccgccggggaagaggaagatcagcagatgtcggaagcgcagatgaaacggacgatgtatgtcacctccaagctcacatccgatggccctccctatgatgttgttacggagacagtccagcaaccagcggtggacgaattatttcaaggagtaagagaagatgcgaaatattacaagcat

>CiVGAT

atggctgcaatattagacaaagttcggtcaagaattgtgtcgttaggaccaacaagtgaagagaaattctcctttgccaagtccagcgaccaaccccactcatcaggaagtgggtgccagggtgatggcgctactaacccaagttccaacagtgtatcgcatccggaacgttcggcctcaggagtggaaaagccgacgattacagcttgggatgccgggtggaatgtctccaatgcaattcagnnnnnnnnnnnnnnnnnnnnnccatacgcagtgttgcacggtggttatctcggacttatactcatcattgtgacagcggtggtttgctgctacaccggcaacatcttaattgactgtctgtacgaaaccagtccttcaggagagcgactgcgggtacgttccacttacgttgacttggcagctcactgttggggaaaacatctgggtggctatctagtaaacgcagcacaattgatagaattattaatgacgtgtgttctttatgtcgtcgtttccggtaacctgatgacaaatagttttccacatggaccaatcagagaagcaggatggtcagtgcttgcatgcctcgttctgttcccttgtatatttcttcgtcacctgagggcggtatcgaggttcagcatggggtgttcggtcgcgcagattgtggttcttggtatcacgatcgtttattgtattacaaagatcaacacatgggcttggagtgagattacgatcagcgtcgatatgaaacagttcccggtctccatcggcgtcatcgtcttcagctatacttcacaaatatttctcccgtcgttagagggcagtatggagaaccgcggggactttcgttccatgttaagttggtcttacgtcgcgtcgtgcgtcacgaaagcgtcgttcgcgcttatatgttttctaacgtggtctaaggatacgaaagacgttgtcactgataacctgcctcctacgctgcgtgcaatgataaacgttctgcttgtggtgaaagctttactttcatatccattgccgtactatcaagctatcgaggtgatggaacaaacaatgttcacgggcgccactggtggctgggggtccctttttggtacgaaacgtcacgcttacggcgagttcaccgatgacacggaacccatcgtacaaagcaccagttttaacaccgacgccacgctggaagatgatacgaacaaacaaagttcttgtccctcctgttattcggcaactggtgatctacaagtgtgggctctagttctgcgagcgggattggtactaggcacactgcttatgggtgtttttatcccccatttcgccttgctaatggggttgactggtagtctcactggaacgtcactcgcttttcttttcccctgcgctttccacctgcaaataaagtggcgggagatgaaatggcgagaaatcgggctcgatgttttcatttttatctcggggaccgtatgcggcatcacaggaatctacttttccattcaaggcctgtacgaggtctataacccgtcccaactctccaactctttgggggccgtaaatggaacatccaacgtcacacacaccgctcttatagctcccggtttctttccagagtttccacttcaaccagacttagacctcccggctttccccgaggaagacttagacctcccggctttccccgaggaaccttttctctcagacaactcgccaacatctggaaacaagaagacaaatgacgacacgatgacgtacctccaggaaagtgacgtcatagacctggtgccacctccacaattaccagacagcgccaaaaccagtcgaagatcagaagccgagcaccttcgtaaacgaaga

>CiVACHT

atggacgtttgtagacagtacggtaacatcgtggccacctttgcttatagttgcattaataaacttagagatttaactgcacctctacgtgaccggatcggagatggaaaatctcagcggaaactcgtgcttgtgatcgtttgtgtagcactactgcttgacaacatgctgtacatggttattgttccaatcataaccgaatatttcaataaaaacgaaacaaagtccgcagtgctttcaacaacatcgcaaataatgaatgactttatcagtaccccatacaccaataacgtcacaccagcagcacttcaatctcatctacaaacttctggacctgacgaagacggtactgaagatactttaaccggaatcctgtttgcgtccaaagcaatcgtgcaattaatggcgaatccgtttactggaacctttatagatcgagttggatacataaagccattaactttggggctgatggttatgtttttatccacagcgttgtttgcttgtgcaaagggctttgctgtattgttcttagctagaagtttacagggtctcggttccgcactagccgatacagcatcccttgggctcattgccgatcgtttccaagatgaagcagagagatcaaaagcgcttggcctcgctctcgcatttatatcatttggcagtttggttgcgccgccatttggtggaattctctatcaatttgccggtagagagtggccgttcctcattctcgcgtttgtgtgcttgatagacgccatgcttcttttattggtgcagatcccaaaagaggatgaaacaaaagcgaaagctggtcacctgccagttggtacaccgatctataaactttttattgacccttatatagcagtgatagccgctgcgctcatggcagctaactttcctctcgcatttttagaacctacaatcgccaagtggatgcacgaaaccatgggttcctctaaatggcaaattggtttagtttggttgcccgcgttcctgccccatattttgggtgtttatctaactgtacgtctatcagtcaagtacttccgcttccaatggctttatggagcaattggcttgttactgattggtgtctctacagctgctgtccccacttgtcacacctacgggattctaatgattccacttgctatcatgtgttttgggattgctctgatcgatacagctttactaccaaccatggccttcctggttgatgttcgtcacactagtgtttacggaagcgtttacgcaattgttgatatctcgtattctgtcgcgtactcactcggtcctatactagccgggcaagcagtccagaaaattggatacttaaaaatgaatgtggccattggcttggcaaatatgcttttttcacctttgttgatctttctgcgagaagtgtatgattggaaacctgataagagtgagagagctgttcttattgaggaaccaacggttatttctgactgtgaatctccagaaagtaacgtttctgaggtacaaaaacaacttccgactcagtctgataattcactgtacgtggttcaacgcccaaggactcgccaaggcagcgtcgtatctagtgaagatgatgaaagtccagatataccaaaccgacgattcagtaaaactgacgcattaaaaatagcgaatcatccgtcacatgtatcaaaatacaacgttctatcagctcgagaagaaacgttcaaaagcagaccgagacctcaaccaagaataaagacatcgatgaagaaatcagcagaacaacatccgagtggtcgatataacacaactgtgttgaatggagaaaaccaagttcacacaaatgacatcagtaacaagtataacacgtcaactctcacaactccgctgtcaaagggagagtttactgcacttgcagaaagtggtttagaagttaggaatccatgttttgagaacgatggacatatatatcaacgaaaa
